# Survival analysis in gastric cancer: a multi-center study among Iranian patients

**DOI:** 10.1186/s12893-020-00816-6

**Published:** 2020-07-13

**Authors:** Atefeh Talebi, Afsaneh Mohammadnejad, Abolfazl Akbari, Mohamad Amin Pourhoseingholi, Hassan Doosti, Bijan Moghimi-Dehkordi, Shahram Agah, Mansour Bahardoust

**Affiliations:** 1 Colorectal Research Center, Iran University of Medical Center, Tehran, Iran; 2 Unit of Epidemiology and Biostatistics, Department of Public Health, University of Southern Denmark, Odense, Denmark; 3 Gastroenterology and Liver Diseases Research Center, Research Institute for Gastroenterology and Liver Diseases, Shahid Beheshti University of Medical Sciences, Tehran, Iran; 4 Department of Statistics, School of Health, Macquarie University, Sydney, Australia; 5 Department of Health System Research, Research Institute for Gastroenterology and Liver Disease, Shahid Beheshti University of Medical Sciences, Tehran, Iran; 6 Department of Epidemiology, School of Public Health, Iran University of Medical Sciences, Tehran, Iran

**Keywords:** Gastric cancer, Survival analysis, Extended cox, Frailty, Akaike information criterion

## Abstract

**Background:**

Gastric cancer (GC) has been considered as the 5th most common type of cancer and the third leading cause of cancer-associated death worldwide. The aim of this historical cohort study was to evaluate the survival predictors for all patients with GC using the Cox proportional hazards, extended Cox, and gamma-frailty models.

**Methods:**

This historical cohort study was performed according to documents of 1695 individuals having GC referred to three medical centers in Iran from 2001 to 2018. First, most significant prognostic risk factors on survival were selected, Cox proportional hazards, extended Cox, gamma-frailty models were applied to evaluate the effects of the risk factors, and then these models were compared with the Akaike information criterion.

**Results:**

The age of patients, body mass index (BMI), tumor size, type of treatment and grade of the tumor increased the hazard rate (HR) of GC patients in both the Cox and frailty models (*P* < 0.05). Also, the size of the tumor and BMI were considered as time-varying variables in the extended Cox model. Moreover, the frailty model showed that there is at least an unknown factor, genetic or environmental factors, in the model that is not measured (*P* < 0.05).

**Conclusions:**

Some prognostic factors, including age, tumor size, the grade of the tumor, type of treatment and BMI, were regarded as indispensable predictors in patients of GC. Frailty model revealed that there are unknown or latent factors, genetic and environmental factors, resulting in the biased estimates of the regression coefficients.

## Background

GC is the 5th leading cause of cancer-related death in spite of its global decrease in incidence and mortality according to GLOBOCAN 2018 data [1]. Despite advances in diagnostic, therapeutic, and screening methods, the mortality rate has not significantly decreased worldwide. In the Iranian population, the prevalence of this cancer is also increasing as it threatens the health of the population [2]. The survival rate of GC is rather low and often the tumor is not diagnosed until an advanced stage that the cause is related to clinicopathological factors [3]. Indeed, malignancy is frequently identified by a variable but poor overall prognosis, particularly in the late clinical stages. The clinicopathological features of GC patients are very imperative in choosing the right therapeutic strategy that can improve patient survival. In addition, diagnosis and treatment of GC considerably depend on prognostic factors and variations of survival over time [4, 5].

Since identifying the prognosis risk factors for GC patients is extensively important, researchers are interested in the survival time until the occurrence of an event in epidemiological and other data [6]. The most reasonable condition in Cox regression is the proportional hazard (CPH) assumption, which is applied in a short-term follow-up. If the proportional assumption does not hold, the results from a CPH model are misleading, and alternative modelling strategies should be carried out [7]. In a common phenomenon in clinical research, time-varying covariance occurs when a given covariate changes over time during the follow-up period that is called an extended Cox model. Indeed, the main characteristic of data with time-dependent covariates is the survivor function for any individual depending on time and the baseline hazard function [8]. The frailty model, the random component, has been designed to account for variability, and it has been used when there is at least one unaccounted predictor in the model [9]. This model assumes that events (e.g., death) happen earlier for individuals who are more frailty. Some factors, including hereditary, genetic characteristics, growth, and living environment, are effective in the caused differences among the patients. When the assumption of proportionality does not hold, applying the CPH regression leads to a biased estimation and underestimation of variance of the parameters [10].

Other studies have been done on survival analysis such as parametric, artificial neural network, Bayesian and parametric, multi-state, and Cure models in GC [11–13]. Viduz et al. surveyed the frailty multi-state model on advanced GC data from the Agamenon National Cancer Registry [14]. Lu et al. performed Cox regression model and log-rank test in patients older than 80 years who underwent radical gastrectomy for primary GC from 2000 to 2012 [15]. Ghadimi et al. analysed the survival rate of the gastrointestinal patients by parametric models such as log-normal, log-logistic, Weibull, and exponential model using with and without frailty, then Akaike information criterion (AIC) was regarded to evaluate the models [16]. Faradmal et al. applied Cox and Frailty models in Breast cancer data and then compared them with the Concordance index [13].

To the best of our knowledge, this is the first multi-center study that investigates the main prognostic factors of GC in Iranian through applying several survival analysis models. The multi-center study is designed to survey multiple cities in Iran. The sample size (*n* = 1695) is large enough to detect a wide range of associations with adequate statistical power.

We aimed to evaluate the effect of important variables on the survival rate of GC patients who registered at three centers in Iran during 2001–2018, using Cox regression and two semi-parametric models.

## Methods

### Patient characteristics

In this study, we included 1695 patients who were diagnosed to have GC and were registered to three separated medical centers in Iran, Rasoul Akram hospital (2013–2018), Taleghani hospital (2003–2007), and Fars province in southern Iran (2001–2006) during 2001–2018. The project was approved in Ethic Committee of Iran University of Medical Sciences (ethical code: IR.IUMS.REC. 1397.481).

- The right-censored data at Rasoul Akram hospital of Tehran represented a historical cohort of 346 GC patients, who registered from September 2013 to November 2018. Demographic and clinical characteristics of patients were obtained by checklists of patient’s records.
- The right-censored data of Taleghani hospital revealed a retrospective review of 746 GC patients who enrolled in the study from February 2003 until January 2007. The patient’s information was gathered by checklists of patient’s records [17].
- The data of the cancer registry of Fars province center demonstrated a historical cohort study of 603 GC patients from March 2001 to March 2006. Prognostic factors of GC and patients’ vital status were collected in March 2006 [18].

The outcome variable was considered as time (months) elapsed since the cancer diagnosis until death. Some important clinical variables containing tumor size, number of involved lymph nodes, distant metastasis, histology, type of treatment, and demographic variables such as age, gender, marital status, education, BMI, and smoking situation were included in the aforementioned models.

### Statistical analysis

Kaplan-Meier, named as the product limit estimator, was used to estimate survival function. In the first step, the Univariate CPH model was performed to find the important factors of GC, chosen variables with *P* < 0.2 in the Univariate analysis were subjected to multivariable regression analysis with *P* < 0.05. The Cox model is a very useful approach to survival analysis. On the other hand, when the assumption of proportionality does not satisfy, the outputs might be misleading, and then other different models should be used [7]. In the time-varying variables, the effects of some predictors depend on the time that is called time-dependent variables, an extended version of the CPH model [19]. The proportional hazard (PH) assumption was assessed using Schoenfeld residuals, and then an extended CPH model was fitted to the data [7]. Since several substantial factors such as genetic or environmental factors were not reported in these data, there was evidence of unmeasured heterogeneity among the patients, so a frailty model was applied [20]. In the frailty model, an unobserved multiplicative effect was considered on hazard function by presuming a g(α) distribution with the unit mean and unknown variance of θ. In the third step, gamma distribution was performed as a frailty component in the Cox model [10]. Finally, the evaluation of models was performed based on the AIC. The significance level for the statistical test was 0.05. The Stata-13 and R-3.2.2 were applied for all statistical analysis.

## Results

In this study, medical files of 1695 GC patients were retrospectively reviewed. 949 (56%) were male, and 746 (44%) were female. The mean age of patients at diagnosis was 60.28 ± 12.93, and the follow-up time was 18.79 ± 16.67 months. The overall median survival rate of 1695 patients was 13.2 months. Eight hundred four (47.4%) of patients were censored, and 891 (52.6%) of patients died at the end of follow-up. The characteristics and pathological features of all the GC patients are presented in Table 1.

**Table 1:** Demographic and pathological characteristics of GC patients

Demographic Characteristics	Censored (***n*** = 804)	Dead (***n*** = 891)	Total (***n*** = 1695)
	Number Percent	Number Percent	Number Percent
**Age (year)**			
< 60	397 49.4	390 43.8	787 46.4
> 60	403 50.1	501 56.2	904 53.3
**Sex**			
Male	471 58.6	478 53.6	949 56
Female	333 41.4	413 46.4	746 44
**Marital status**			
Unmarried	50 6.2	51 5.7	101 6
Married	749 93.2	835 93.7	1584 93.5
**Education**			
Illiterate	220 27.4	125 14	345 20.4
Primary	199 24.8	199 22.3	398 23.5
University	58 7.2	88 9.9	146 8.6
**Family history**			
Yes	201 25	263 29.5	464 27.4
No	563 70	576 64.6	1139 67.2
**BMI**			
< 18.5	92 11.4	112 12.6	204 12
18.6–24.9	394 49	453 50.8	847 50
> 25	115 14.3	109 12.2	189 11.2
**Smoking status**			
Yes	326 40.5	431 48.4	757 44.7
No	439 54.6	392 44	831 49
**Tumor type**			
Adenocarcinoma	384 47.8	427 47.9	811 47.8
Lymphoma	73 9.1	44 4.9	117 6.9
Other	93 11.6	59 6.6	152 9
**Lymph node**			
N1	139 17.3	136 15.3	275 16.2
N2	209 26	233 26.2	442 26.1
N3	39 6.1	37 4.2	86 5.1
**Metastasis**			
Yes	168 20.9	401 45	569 33.6
No	451 56.1	403 45.2	854 50.4
**Treatment**			
Surgery	437 54.3	470 52.7	907 53.5
Surgery+ chemotherapy	245 30.5	230 25.8	475 28
Chemotherapy+ radiotherapy	122 15.2	191 21.5	313 18.5
**Tumor size**			
T1	118 14.7	107 12	225 13.3
T2	229 28.5	208 23.3	437 25.8
T3	94 11.7	156 17.5	250 14.7
**Grade**			
Well	286 35.6	300 33.7	586 34.6
Moderate	194 24.1	311 34.9	505 29.8
Poorly	124 15.4	110 12.3	234 13.8
Undifferentiated	165 20.5	126 14.1	291 17.2

**Fig. 1:**
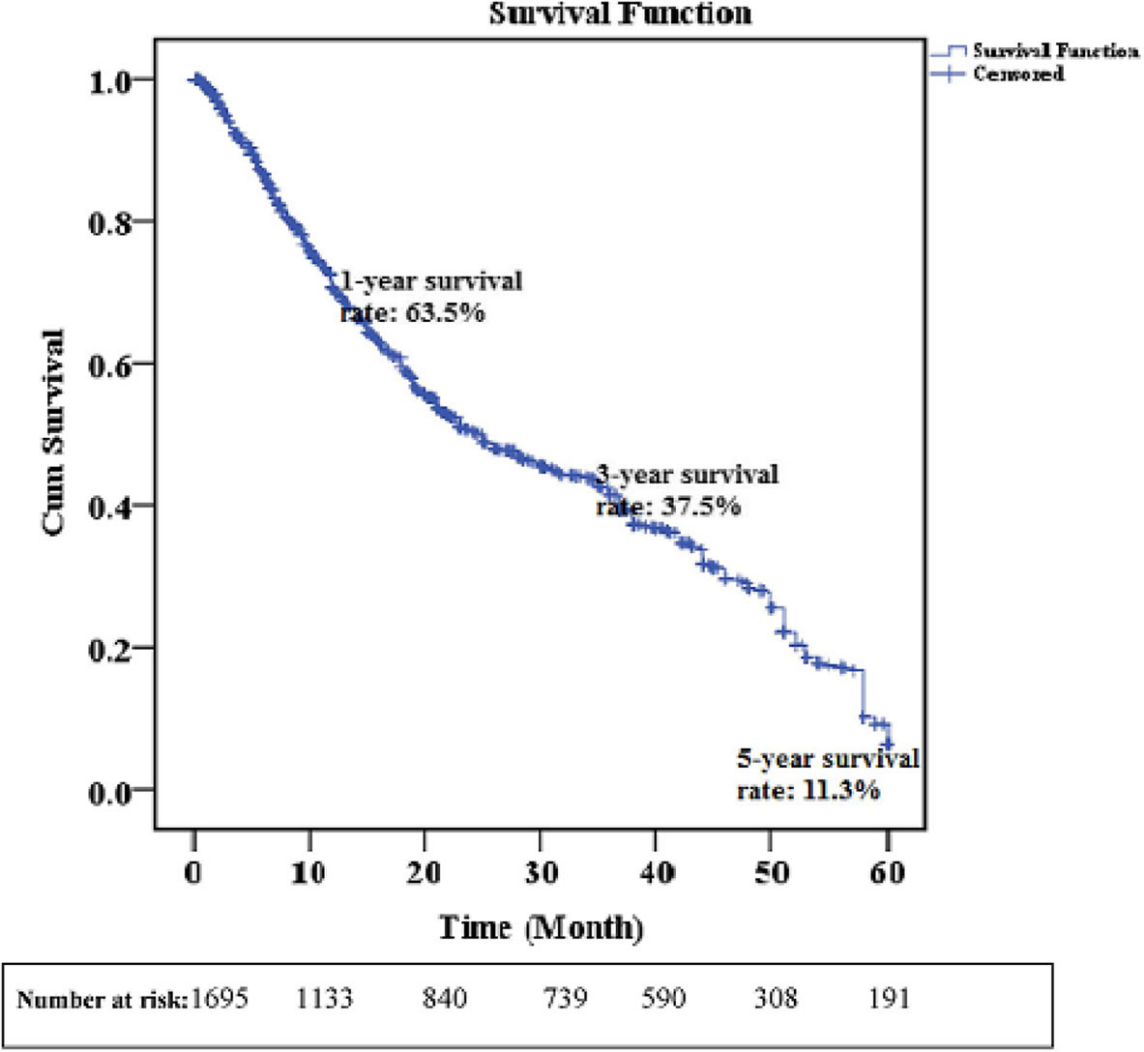
Kaplan–Meier curve, 5-year survival rate

Based on Kaplan–Meier estimates, the 5-year survival rate was assessed 11.3%, while one-year and three-year survival rates were 63.5 and 37.5%, respectively (Fig. 1).

The test based on Schoenfeld residuals revealed that some variables, including the size of the tumor, the grade of the tumor, and BMI (*P* < 0.05), were violated from the PH assumption (Global *P* = 0.04). The results of the CPH were provided in Table 2. The table showed that age, sex, smoking, BMI, type of tumor, involved lymph node, metastasis, type of treatment, tumor size, the grade of the tumor are significant in the univariate Cox model (*P* < 0.15). Moreover, the output of the multivariate Cox model revealed that age, BMI, type of treatment, tumor size, and grade of the tumor are statistically significant (*P* < 0.05).

**Table 2:** Univariate and multivariate analyses of CPH model for prognosis in GC patients

	Univariate	Multivariate
	HR P	HR P
**Age (year)**		
< 60	Ref -	
> 60	1.006 0.03	1.01 0.018
**Sex**		
Male	Ref^* -^	
Female	1.22 0.004	
**Marital status**		
Unmarried	Ref -	
Married	1.03 0.84	
**Education**		
Illiterate	Ref -	
Less than diploma	0.94 0.6	
University	0.82 0.17	
**Family history**		
Yes	Ref -	
No	1.09 0.23	
**BMI**		
< 18	1.66 < 0.001	2.45 < 0.001
18–25	0.98 0.87	1.08 0.65
> 25	Ref	
**Smoking status**		
No	Ref -	
Yes	1.22 0.004	
**Tumor type**		
Other	Ref -	
Adenocarcinoma	0.8 0.11	
Lymphoma	0.6 0.01	
**Lymph node**		
N1	Ref -	
N2	1.4 0.002	
N3	1.27 0.19	
**Metastasis**		
No	Ref -	
Yes	1.3 < 0.001	
**Treatment**		
Chemotherapy+ radiotherapy	Ref -	
Surgery+ chemotherapy	0.58 0.001	0.79 0.011
Surgery	0.95 0.58	0.85 0.052
**Tumor size**		
T1	Ref -	
T2	1.5 < 0.001	2.43 0.002
T3	3.6 < 0.001	2.77 0.001
**Grade**		
Undifferentiated	Ref -	
Well	0.73 0.004	0.650192
Moderate	0.83 0.088	0.38 < 0.001
Poorly	0.96 0.77	0.68 0.129

*Abbreviations*: *HR*: Hazard Ratio, *Ref* Reference group

A HR of tumor size categories indicated that T2, T3 (larger sizes) had worse prognoses compare to T1 (small size) (*HR* = 1.5; *HR* = 3.6, *P* < 0.001). Moreover, the HR of metastasis was 0.3% more than non-metastasis (*HR* = 1.3; *P* < 0.001). In addition, the HR of death in patients with N2 (N3) was 40% (27%) times more than those with N1. Additionally, the HR of treatment type in surgery plus chemotherapy was 0.21% less than chemotherapy plus radiotherapy that seems to be significant (*HR* = 0.79, *P* < 0.05). Based on the Multivariate CPH model, the HR of death of patients with BMI < 18 (underweight) is 2.45 times more than those with overweight (*HR* = 2.45, *P* < 0.001). Also, the AIC is − 1620.36 in the CPH model. Table 3 showed the results in evaluating PH to apply the extended Cox model and frailty model.

**Table 3:** PH assumption for the extended Cox and frailty models

	PH assumption	Frailty
	HR P	P (rho) HR P
**Age (year)**		
< 60	Ref -	
> 60	1.25 0.08	0.0156 1.32 0.03
**Sex**		
Male	Ref -	
Female	0.91 0.48	0.89 0.38
**Marital status**		
Unmarried	Ref -	
Married	1.88 0.041	1.74 0.07
**Education**		
Illiterate	Ref -	
Less than diploma	0.92 0.58	0.97 0.88
University	0.91 0.6	0.91 0.59
**Family history**		
Yes	Ref -	
No	0.84 0.17	0.87 0.3
**BMI**		
< 18	2.7 < 0.001	0.0164 2.6 0.001
18–25	1.17 0.54	1.04 0.81
> 25	Ref -	
**Smoking status**		
No	Ref -	
Yes	1.08 0.52	1.11 0.41
**Tumor type**		
Other	Ref -	
Adenocarcinoma	0.97 0.91	0.94 0.81
Lymphoma	0.89 0.72	0.81 0.49
**Lymph node**		
N1	Ref -	
N2	1.25 0.08	1.21 0.14
N3	0.83 0.43	0.83 0.43
**Metastasis**		
No	Ref -	
Yes	0.95 0.71	0.98 0.86
**Treatment**	< 0.001	
Chemotherapy+ radiotherapy	Ref -	
Surgery+ chemotherapy	0.69 0.001	0.81 0.021
Surgery	0.91 0.05	0.9 0.056
**Tumor size**		
T1	Ref -	
T2	2.37 0.01	0.0158 2.27 0.013
T3	3.04 0.002	2.53 0.008
**Grade**		
Undifferentiated	Ref -	
Well	0.51 0.12	0.43 0.047
Moderate	0.30 0.002	0.26 0.001
Poorly	0.56 0.125	0.54 0.099
		**Variable Coefficient P**
		Variance of Frailty 1.67 0.04

Since the PH assumption was not met for tumor size, age and BMI, CPH model no longer satisfies the PH assumption and therefore an extended Cox model was performed. Furthermore, the Global test demonstrated CPH did not seem to be suitable because of non-proportional hazards (*P* = 0.04). The result of the extended Cox model revealed that the size of the tumor and BMI are time-varying variables (*P* < 0.05 and the AIC is − 1452.07 in this model.

Furthermore, Table 3 indicates the significant variables in the frailty model, including age, tumor size, the grade of the tumor, and BMI (*P* < 0.05). The results showed that the variance of frailty was significantly greater than zero (θ = 1.67; *P* < 0.05). According to our finding, there were latent factors which affect the hazard of death. The AIC in the frailty model is − 412.72, which is larger than − 1620.36 and − 1452.07 in CPH and extended Cox models. These results of comparing AIC in three models indicate that the best models are frailty, extended Cox, and CPH, respectively.

## Discussion

Survival analysis have mostly performed using common statistical methods such as CPH that have shortcomings [2, 21–23]. However, this is the first multi-center study, comparing different survival models to identify prognostic risk factors in GC patients of developing countries. GC is a worldwide cause of cancer death with a low 5-year survival rate in Iran. A number of factors have been identified as predictive prognosis factors until now [24–26]. In the recent study, the CPH, extended Cox, gamma frailty Cox models were fitted to determine the adjusted hazard of GC patients who underwent treatments, surgery, chemotherapy, and radiotherapy. The significant variables in the CPH model were age at diagnosis, BMI, tumor size, type of treatment and grade of the tumor. Additionally, time-dependent variables, the size of the tumor, and BMI with time function g(t) = t were considered in the extended Cox model. Furthermore, the frailty model was chosen as the best model and demonstrated that there are latent factors that affect the hazard of death. The results revealed that age, tumor size, the grade of the tumor, type of treatment and BMI have a direct effect on the hazard of death in GC patients.

The results of CPH and frailty models showed a significant relationship between age over 60 years at the time of surgery and the 5-year survival of patients with GC. Similar to our results, previous studies have reported the relationship between age and the 5-year survival [24, 26]. The main strength of these investigations was the large sample size that was consistent in our study. Whilst a contradictory result of a study indicated that there is not any relationship between older age and patient survival, which could be because of differences in the sample size [25]. A retrospective study revealed how patients over the age of 70 years differ from younger patients in postoperative courses with a focus on the frequency of surgical and medical [27].

The findings of our study showed that both sizes of the tumor and tumor grade in patients at the time of diagnosis in three models were significantly associated with patients’ survival. The size of tumor and tumor grade have been identified as a risk factor for the survival of GC patients, which are compatible with our study [24, 26] while the result of Nasseri et al. demonstrated that there is no relationship between the grade of tumor and GC [28].

The main result of our study showed a significant relationship between the survival of GC patients with BMI < 18, which was consistent with the findings of BMI in the study of Liu et al. [29]. In their study, a 320 cohort study was conducted to survey the effect of BMI and recreational physical activity on GC risk. It can be illustrated due to anorexia, weakness and poor health status of under-weight patients, therefore the variable can be regarded as indispensable prognostic factor. In general, studies of BMI and GC have been restricted. Furthermore, combined surgical treatment and chemotherapy were related to higher survival rate that the result was consistent with several previous studies [30–33]. A meta-analysis was performed to survey the effect of treatment type on GC [33]. Moreover, combination therapy surgery as well as chemotherapy was significant in some cancers such as Hepatocellular carcinoma [34]. However, an inconsistent study presented the survival analysis of GC patients with incomplete data that treatment type was not significant [4].

The frailty model is performed to explain the random variation of the survival function that may exist due to some unobserved genetic prognostic factors such as genetic and other environmental factors. These results are consistent with the results of many studies in this field [16, 35].

Based on the AIC, the frailty model is the best alternative model for the Cox proportional hazard model. This issue is consistent with most studies conducted on GC patients [10, 15, 28].

### Strengths and limitations

The main strength of the present investigation was the multi-center study with a large sample size as well as a lack of missing in the data. The main limitation of this study was the short- term follow-up period. Further, studies with longer follow-up periods may provide more determining evidence regarding the Survival predictors in GC patients.

## Conclusion

The results of this study indicate that the age > 60 years, tumor size, the grade of tumor, type of treatment and BMI < 18 kg/m^2^ are the main prognostic factors in the survival rate of GC patients. In fact, they are reducing the survival rate of GC patients. Also, based on our findings from the frailty model, we might conclude that employing a more intricate statistical model that regards the significant role of latent variables on hazard ratio, including unobserved genetic or environmental factors, would expand the importance of the more analyses.

## Data Availability

The datasets used and analysed during the current study are available from the corresponding author on reasonable request.

## Abbreviations

HR: Hazard rate
BMI: Body mass index
GC: Gastric cancer
CPH: Cox proportional hazards model
PH: Proportional hazards
AIC: Akaike information criterion
